# An examination of the potential benefits of expert guided physical activity for supporting recovery from extreme social withdrawal: Two case reports focused on the treatment of *Hikikomori*

**DOI:** 10.3389/fpsyt.2023.1084384

**Published:** 2023-03-23

**Authors:** Keiko Yokoyama, Tadaaki Furuhashi, Yuji Yamamoto, Maki Rooksby, Hamish J. McLeod

**Affiliations:** ^1^Research Center of Health, Physical Fitness and Sports, Nagoya University, Nagoya, Japan; ^2^Department of Psychopathology and Psychotherapy, Nagoya University Graduate School of Medicine, Nagoya, Japan; ^3^School of Neuroscience and Psychology, University of Glasgow, Glasgow, United Kingdom; ^4^School of Health and Wellbeing, College of Medical Veterinary and Life Sciences, University of Glasgow, Glasgow, United Kingdom

**Keywords:** *Hikikomori*, social withdrawal, outdoor workout, interpersonal sports, physical activity

## Abstract

Extreme and long-term social withdrawal, first described in Japan as *Hikikomori*, has now become a globally recognized mental health problem. Intervention studies severely lag behind epidemiological and phenomenological research. We present two descriptive case reports of Japanese university students with *Hikikomori* who participated in an early phase test of a structured intervention involving physical activities that was developed and facilitated by clinicians and physical education specialists—Human Movement Consultation (HMC). The two recipients (19- and 29-years old at the start of treatment) completed approximately 40 consultation sessions delivered over 3 years consisting of a combination of outdoor workouts (i.e., walking, running, and cycling) and interpersonal sports (e.g., table tennis, badminton, and tennis). Changes in social withdrawal behavior were independently rated from clinical health records using a structured scale (the Glasgow *Hikikomori* Scale; GHS). Behavioral observations and scale data for both cases indicated improvements from pre-treatment levels of social withdrawal. At the end of the intervention, both had returned to normative levels of functioning. Case A returned to university and Case B secured a new job upon the completion of HMC. To help advance our understanding of treatment options, these case descriptions analyze potential change mechanisms in order to understand how HMC can support recovery from extreme social withdrawal. One key observation is that both outdoor workouts and interpersonal sports offer a non-threatening method of enabling *Hikikomori* to engage in interpersonal interactions. Such connections via structured activities may allow the reinstatement of social skills in a graded manner. In addition, an initial focus on physical experiences may help promote psychological and social connectedness without triggering the social fears and challenges that underlie the *Hikikomori* state. The findings from these two cases offer a framework to guide further research and the development of exercise-based interventions for this hidden and often neglected group.

## 1. Introduction

Severe and enduring social withdrawal, referred to by the Japanese term “*Hikikomori*,” was first observed in Japan in the 1990s. Today, it is an important but often overlooked problem (1–5). Cases of prolonged social withdrawal have been reported across countries with diverse cultures and economic characteristics (6, 7): including the United States (8), France (9), Spain (10, 11), and Oman (12), among others. Consistent with a recent proposal for defining the diagnostic criteria for *Hikikomori* (13), the governmental guidelines in Japan suggest that the affected individual remains in social isolation at home for at least 6 months (14, 15). The state of social withdrawal must also be accompanied by significant functional impairment and distress, such as the inability to attend school or work, thereby avoiding meeting expectations that are deemed reasonable by social conventions. Importantly, *Hikikomori* can be distinguished from conditions such as agoraphobia and social phobia where the periods of withdrawal are usually much shorter and the avoidance of social interaction is explicable as a way of managing panic attacks or fear of negative social evaluation. In these conditions if the panic or fear of negative appraisal is addressed, the withdrawal behavior is extinguished. In contrast, the withdrawal pattern seen in *Hikikomori* is marked by a more enduring disengagement from normal social interactions with the aim of “disappearing” from society. As noted, this may be so extreme that the person may not leave their bedroom for many months or years. However, these withdrawal behaviors do show some variation across the natural history of *Hikikomori*. For example, after meeting the initial criterion of withdrawing for longer than 6 months some *Hikikomori* may show tentative re-engagement with the outside world but at a level well below what would be considered developmentally normal. This type of partial recovery from the *Hikikomori* state is the point at which most psychosocial intervention options are currently attempted.

While it has been observed that *Hikikomori* can co-occur with other mental health conditions such as mood disorders (8), autism (16), and the prodromal phase of psychosis (17), for the majority extreme social withdrawal is the only significant presenting issue. Therefore, co-morbidity is not currently regarded as a defining clinical profile for diagnosis (1). Research focused on understanding *Hikikomori* has been evolving with growing international recognition, and it is currently characterized as a sociocultural mental health phenomenon or syndrome, rather than a mental disorder. Although studies on the etiology, diagnosis, and prevalence of *Hikikomori* have proliferated, research on viable interventions is lagging behind.

Given that a critical feature of *Hikikomori* is extreme aversion and sensitivity to interacting with others, it is likely that therapy approaches that are indirect and non-threatening will be more acceptable. Furthermore, using therapies that involve physical activity may support recovery of both physical and mental health. It is well-recognized that “(r)egular physical activity is proven to help prevent and manage noncommunicable diseases such as heart disease, stroke, diabetes, and several cancers. It also helps prevent hypertension, maintain healthy body weight, and can improve mental health, quality of life and well-being” (18). Moreover, the National Institute of Health and Nutrition in Japan suggests the importance about physical activity to maintain and improve of mental health (19). The sedentary lifestyle of *Hikikomori* is likely to elevate the risk of hypertension and prehypertension (20), which can hasten the development of cardiovascular diseases. Moreover, in terms of mental health, interventions involving physical activity have shown efficacy in improving issues such as depressive symptoms (21, 22), or for recovery of the sense of body awareness (23) and a sense of identity (24). Various types of physical activities have been reported as beneficial, including aerobic exercise (25), yoga (26), and dance (23). A single case study of a youth with *Hikikomori* has reported that regular jogging was effective for increasing the cerebral hemodynamics, while reducing social withdrawal symptoms (27). In addition, engagement in team sports, such as soccer, badminton, and volleyball have been endorsed in a support group for *Hikikomori*, with the rationale that team sports provide an opportunity for social communication and connection (28). Thus, existing studies support physical activities as a potential option to treat *Hikikomori* symptoms, including its core issue, social withdrawal. We used a descriptive case study approach to help address the current knowledge gap regarding the benefits of physical activity for extreme social withdrawal. Specifically, we aimed to explore the types of physical activities that may be suitable for reducing *Hikikomori* behaviors as a way of expanding the range of viable treatment options for *Hikikomori*.

To date, most research on *Hikikomori* has been focused on epidemiology and phenomenology rather than treatment and so there is very little known about the effectiveness of psychosocial approaches (e.g., CBT) that are commonly used to help people recover from mental health and wellbeing problems. Because the interpersonal demands of many psychotherapy approaches may be challenging for people showing extreme social withdrawal we decided to explore a physically-oriented therapy approach that we expected could be well-tolerated. In this study, we present case reports of two university students with *Hikikomori*, who received a novel intervention involving physical activities—human movement consultation (HMC). The primary aims of the HMC are to support the recovery of physical fitness, circadian rhythms, body awareness, and sociality. The approach is highly flexible and challenges to increase social engagement and interpersonal performance only proceed at a pace that the recipient can tolerate. This is crucial because avoidance of perceived societal demands for prescribed social behaviors is thought to be a key part of the etiology of the *Hikikomori* state. The HMC was developed by the research team as a part of the student counseling services at a Japanese university, by collaboration between the faculty members in the physical education and psychiatry departments. The HMC aimed to provide flexible intervention options selected from two groups of physical activities: outdoor activities and interpersonal sports. Given the differences in the nature of these two activities, we anticipated that they would each offer unique and meaningful options to the recipients. In turn, the two types of activities allowed the research team to adjust and adapt the programme for the clients' recovery process and with any change in their core issues. Consequently, by recording the activity choices made, we could explore whether types of activity showed any correspondence with each person's stage of recovery. We were particularly interested to explore any changes in activity preference as the recovery process unfolded.

## 2. Materials and methods

### 2.1. The human movement consultation (HMC) program

#### 2.1.1. Procedures and principles

The HMC was provided by faculty members at the university specializing in physical education, hereafter referred to as “counselor(s).” The counselor held a Ph.D. in Psychology and was a physical education instructor with special expertise in designing and delivering physical activity programs for vulnerable and high needs populations. The two clients investigated in this study were both male Japanese students with *Hikikomori* who agreed to participate in the HMC following referral by their psychiatrist (TF). The students continued to receive clinical support and independent monitoring from their psychiatrist throughout the duration of the HMC trial.

An important principle of the HMC was to incorporate the interests and preferences of the recipient and to avoid imposing strict or rigid rules. Individuals affected by *Hikikomori* are fearful of and averse to external pressures such as those specified in social rules. Activity types for the sessions were not pre-determined by the intervention team; instead, the activities for subsequent sessions were set at the end of the preceding session by the client, with input provided by the counselor if required. The selection of activity types were sometimes suggested (rather than instructed) by the counselor, and some other sessions required review of the plan due to factors relating to the weather. The standard session frequency was once every 2 weeks, but this was not fixed and could be changed based on the individual needs and preferences of the client. The counselor and client discussed the date of next consultation at the end of each session, to suit the client preference and availability. The planned duration of each HMC session was set at 1 h, but this was also flexible depending on the nature of the activity and clients' preferences. The outdoor workout sessions tended to be longer, as it involved the travel time for starting and ending the session at the university. The configuration of participants in each HMC session, was usually a dyadic form (i.e., counselor and client), but sometimes the dyads would join an external group activity or an additional member joined them to form a triadic interaction. These variations in membership were introduced to reflect the client's readiness for wider social contact and progress via consultation with the psychiatrist, and aimed at introducing variety in social communication opportunities. Most conversations between the client and counselor took place around the physical activities undertaken in the sessions. An important principle of the HMC was that the counselor would not mention *Hikikomori*, unless the client voluntarily raised this topic.

#### 2.1.2. HMC activity types

Two types of HMC activities were offered: (1) outdoor workouts (e.g., walking, running, and cycling), and (2) interpersonal sports (e.g., tennis, table tennis, and badminton).

**Figure 1:**
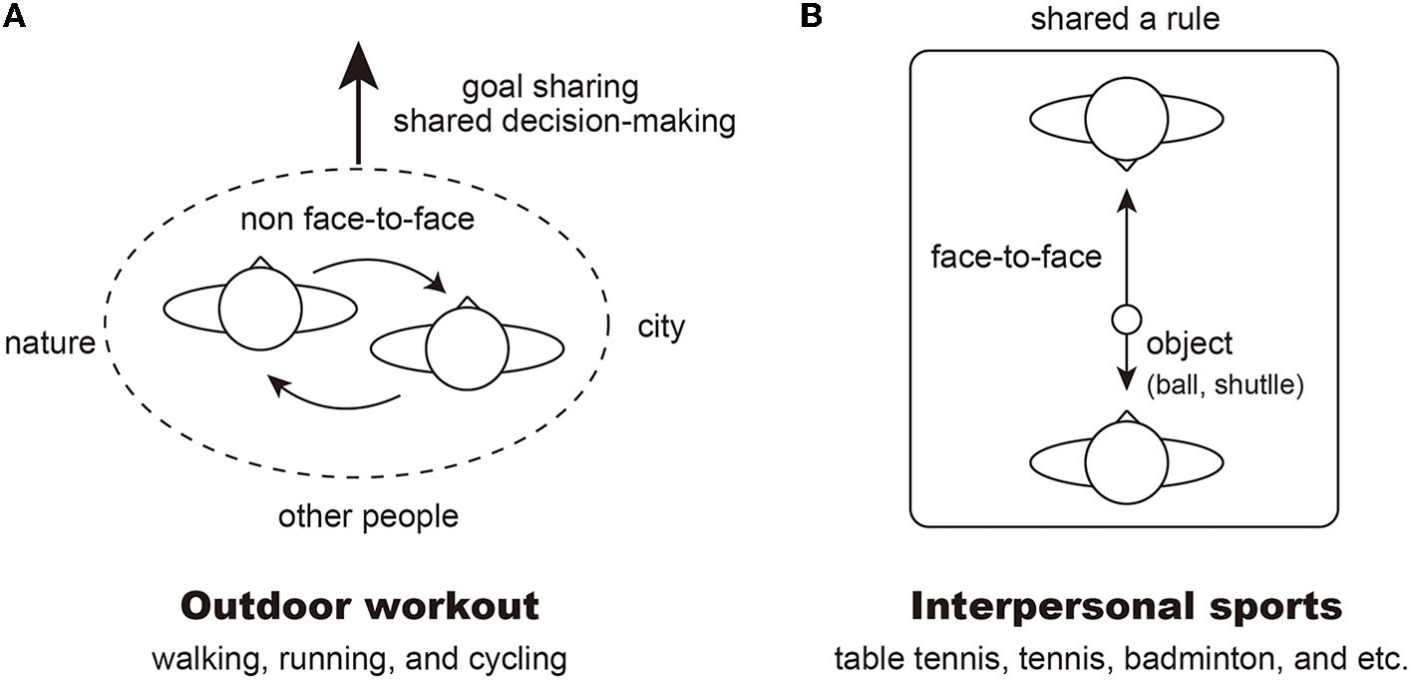
Two types of activities offered by human movement consultation (HMC). **(A)** Outdoor workout. **(B)** Interpersonal sports.

As shown in Figure 1A, outdoor activity, such as walking, running, and cycling involved mobility, and are characterized by rhythmic and periodical movements. Such movements facilitate the regulation of cardiac rhythm and breathing, and were expected to help improve the cardiopulmonary functioning of the clients included in this study. Further, the nature of such outdoor activities would mean that participants' eye gaze are directed toward the direction of travel, rather than directly at each other. Given the nature of *Hikikomori* symptoms, this socio-spatial focus of joint attention would seem likely seen as acceptable for them. Previous studies have shown that humans tend to produce synchronized rhythmic movements when performing activities together with others, and that this synchronization occurs spontaneously (29, 30). Thus, rhythmic movements involved in such outdoor workouts may provide *Hikikomori* patients a chance to experience social interaction without having to rely on cognitive processes needed to manage joint social activities. In addition, the therapeutic activity of a “walk and talk” intervention in an outdoor environment brings benefits such as promoting a collaborative stance that invites clients to express their preferences and choices and act together on them (31). Moreover, outdoor workouts involving mobility offer an opportunity for sharing goals and decision-making processes, such as choice of destination and route during activity with another individual, the counselor. *Hikikomori* individuals often show a tendency to avoid making decisions or making an impact on others. Optional and non-consequential decision-making opportunities provided by locomotive actions during the HMC programme may gently and safely encourage them to process decision making situations with guidance of the counselor. Through the outdoor workout in the HMC, and with support from the counselor, *Hikikomori* may be able to take up the opportunity to be an active agent in the social world, instead of passively “watching the world go by.”

As shown in Figure 1B, interpersonal sports activities of a reciprocal nature (e.g., table tennis, badminton, and tennis) were offered to the clients as an interpersonal alternative to locomotive activities in purpose-built facilities. Unlike the outdoor sessions, these sessions introduced face-to-face interaction to the clients. In addition, participation in these sports entailed non-verbal and symbolic exchange of social quality, such as turn-taking via a ball. We considered that such sports may offer an ideal context for the clients to participate in non-verbal communication without the socio-cognitive pressure that may be felt during psychotherapeutic sessions. Another aspect of these sessions is that they could, by their nature, distinguish a winner from a loser much more explicitly than the activities in the outdoor sessions. The pain of accepting failures, and the fear of not achieving the socially desirable outcome, are the core difficulties of the *Hikikomori* population. Thus, the option of interpersonal sports could be seen as particularly challenging or painful for the clients, because of the symbolic meaning associated with these activities; on the other hand, they also offered an opportunity to take on the challenge as deemed suitable during their recovery process, with support of the counselor. The counselor applied flexibility and sensitivity in adapting the formal rules of the sports featured in the sessions. For example, they may set a shared goal (e.g., keeping a number of rallies in table tennis, without either player dropping a ball), instead of following the formal rule or competing against each other.

### 2.2. Assessment of social withdrawal

Because of the challenges of getting people who are severely withdrawn to complete self-report measures, we developed an observer-based measure for use in this study and in other clinical practice settings. The Glasgow *Hikikomori* Scale (GHS) is an observer rated measure that assesses the extent of social withdrawal across three subscales: Daily Life and Self Care (four items), Occupational Role (five items), and Social Interaction (six items) (32). The correspondence between scores on each subscale and the operational definition of each observed level of functioning are summarized in Table 1. In the study, the psychiatrist (TF) assessed the *Hikikomori* symptoms of the clients approximately every 6 months during the HMC period, based on the rating of case notes retrospectively.^1^ Because this scale allows observational assessment of withdrawal symptoms, without requiring direct input from the *Hikikomori* person the trajectory of withdrawal symptoms could be independently tracked and recorded by the psychiatrist providing care.

**Table 1:** Typical functioning of social withdrawal level corresponding each subscale of Glasgow *Hikikomori* Scale (GHS) (32).

**GHS subscales**	**Level**	**Typical function**
Daily Life and Self Care	4	Spontaneously engages in a social life and activities of daily living (e.g., grooming, eating, sleeping, etc.)
	3	Engages in a social life and activities of daily living, but only with the prompting and support of others (e.g., parent/carer)
	2	Displays restricted and diminished social life and daily living skills despite prompting and encouragement from others (e.g., parent/carer)
	1	Engages with only the minimum daily activities and tasks (e.g., hygiene, eating, sleeping, etc.)
Occupational Role	5	Spontaneously and independently maintains age appropriate roles (e.g., work or study)
	4	Maintains age appropriate roles but only with prompting and support from others (e.g., parent/carer)
	3	Engages in age appropriate roles that are well below their ability and expected attainment
	2	Only engages in age appropriate roles that are well below their ability and expected attainment with prompting and support of others (e.g., parent/carer)
	1	No engagement in age appropriate roles (e.g., work or study)
Social Interaction	6	Spontaneously seeks out direct contact with others, both familiar and unfamiliar
	5	Engages in limited direct (e.g., face to face) and indirect social contact (e.g., online, via mobile social media apps, mobile phone)
	4	Forms the intention to seek social interaction outside of home but has abandoned attempts at making social contact with others
	3	Engages in infrequent social interactions (less than one contact per week) by any means (including with family members and/or online/social media)
	2	Has no social interaction at all outside of home and no interaction with family members (social interaction only online/via social media)
	1	Has no social interaction including via family members and indirect social contact in cyberspace (only engages in social uses of the Internet as an observer)

### 2.3. Participants

The two participants (Cases A and B) were both male Japanese students with *Hikikomori*. At the time of treatment these two participants had shown some recovery from the most severe phase of *Hikikomori* in that they were able to go outside of their houses occasionally. Nevertheless, their symptoms and history meant that they met the criteria for a diagnosis of *Hikikomori*; neither of them was capable of communication with members of the general public, nor were they able to attend university or engage in part-time employment. Their symptom profile, presenting partial capacity to go outside, provided a chance for the participants to engage with the HMC, while allowing the research team an opportunity to explore feasibility of the novel approach with them. They agreed to participate in the HMC as part of the treatment plan following recommendations by their psychiatrist.

Case A was a Japanese 19-year-old male undergraduate student. At the time of starting participation, he had been absent from the university for a year, but had made some recovery from his withdrawal symptoms and was able to attend the bi-weekly psychiatric consultation with parental support. He reportedly spent most of his time playing internet games in his room, with his day and night schedules reversed. The psychiatric assessment suggested diagnosis of avoidant personality disorder with communication difficulties, rather than a developmental disorder.

Case B refers to a Japanese 29-year-old male who was a graduate student at the start of receiving the HMC. He began the HMC intervention approximately 3 years following the onset of *Hikikomori*. At that time, he was able to secure part-time work, go out, exercise, and attend his biweekly appointments with his psychiatrist. Nonetheless, he was not able to engage in his studies.

These two participants provided written informed consent to participate in this study. They also gave the formal consent for their experiences to be written up as a case study. Personal information relating to the two cases has been modified to ensure the participants' privacy and anonymity. The research protocol of this study was approved by the institutional ethics committee of the Research Center of Health, Physical Fitness and Sports, Nagoya University, Japan (approval number: 29-06), and the study conformed with the principles stated in the Declaration of Helsinki.

## 3. Results

### 3.1. Case A

#### 3.1.1. The HMC sessions

Case A underwent 39 HMC sessions including an assessment interview which amounted to about 42 h of consultation (Mean ± SD = 66.1 ± 15.4 min/session; Min = 60 min/session; Max = 120 min/session; note: a recording unit applied for session duration was 30 min, with durations in between rounded up or down depending on whether the minutes recorded was greater than the midpoint of 30, i.e., 15. E.g., a 70 min session was recorded as 60 min, whereas a 80 min one as 90 min.). The period between the initial and final sessions comprised a total of 826 days (2 years and 96 days). The mean interval between consecutive sessions were 21.7 days (SD = 14.1 days; Min = 4 days; Max = 61 days). Table 2 provides the details of the HMC sessions: session dates, interval days between sessions, session duration, activity type, and location.

**Table 2:** HMC details of Case A (39 sessions).

**Number**	**Date**	**GHS score**	**Interval**	**Duration**	**Activity type**	**Location**	**Member**
	**(month-year)**	**Sum (DS, OR, SI)**	**(days)**	**(min)**			
	Oct-X	6 (2, 2, 2)					
1	Nov-X		–	60	Interview	Univ.	H, C
2	Nov-X		25	60	Walking	Forest park	H, C
3	Jan-X+1		42	90	Walking	Temple	H, C
4	Jan-X+1		13	60	Table tennis	Gym in Univ.	H, C
5	Feb-X+1		35	60	Playing catch-a-ball	Park around Univ.	H, C
6	Mar-X+1		14	60	Cycling, Playing catch-a-ball	Lake park	H, C
7	Mar-X+1		14	90	Cycling, Playing catch-a-ball	Lake park	H, C
	Apr-X+1	7 (2, 2, 3)					
8	Apr-X+1		14	90	Cycling, Badminton	Lake park	H, C
9	Apr-X+1		14	60	Cycling, Walking	Forest Park	H, C
10	May-X+1		15	120	Cycling, Walking	Forest Park	H, C
11	May-X+1		14	120	Cycling, Walking	Forest Park	H, C
12	Jun-X+1		14	60	Walking, Running	Forest Park	H, C
13	Jun-X+1		21	60	Walking, Running	Forest Park	H, C
14	Jul-X+1		28	60	Table tennis	Gym in Univ.	H, C
15	Sep-X+1		42	60	Walking, Running	Around Univ.	H, C
16	Sep-X+1		14	60	Walking, Running	Around Univ.	H, C
17	Oct-X+1		22	60	Walking, Running	Forest Park	H, C
	Oct-X+1	7 (2, 2, 3)					
18	Oct-X+1		14	60	Walking, Running	Forest Park	H, C
19	Nov-X+1		19	60	Walking, Running	Forest Park	H, C
20	Nov-X+1		5	60	Walking	Around Univ.	H, C
21	Nov-X+1		11	60	Walking, Running	Forest Park	H, C
22	Dec-X+1		14	60	Table tennis	Gym in Univ.	H, C
23	Dec-X+1		7	60	Walking, Running	Forest Park	H, C
24	Jan-X+2		21	60	Walking, Running	Forest Park	H, C
25	Feb-X+2		36	60	Walking, Running	Forest Park	H, C
26	Feb-X+2		6	60	Table tennis	Gym in Univ.	H, C
27	Mar-X+2		26	60	Table tennis	Gym in Univ.	H, C, O
28	Apr-X+2		14	60	Walking, Running	Forest Park	H, C, O
	Apr-X+2	8 (2, 3, 3)					
29	Apr-X+2		18	60	Walking, Running	Forest Park	H, C, O
30	May-X+2		28	60	Walking, Running	Forest Park	H, C, O
31	Jun-X+2		42	60	Table tennis	Gym in Univ.	H, C
32	Jul-X+2		13	60	Walking, Running	Forest Park	H, C, O
33	Sep-X+2		61	60	Table tennis	Gym in Univ.	H, C
34	Sep-X+2		10	60	Table tennis	Gym in Univ.	H, C
	Oct-X+2	8 (2, 3, 3)					
35	Nov-X+2		49	60	Walking, Running	Forest Park	H, C
36	Nov-X+2		20	60	Walking, Running	Forest Park	H, C
37	Jan-X+3		57	90	Walking	Forest Park	H, C
38	Feb-X+3		7	60	Walking, Running	Forest Park	H, C
39	Feb-X+3		7	60	Walking, Running	Forest Park	H, C
	May-X+3	8 (2, 3, 3)					
Mean ± SD			21.7 ± 14.1	66.1 ± 15.4			
Min			4	60			
Max			61	120			
Sum			826	2520 (42 h)			

The items of Interval and Duration indicates the days between two consecutive sessions and duration of HMC, respectively. H, C, and O indicate *Hikikomori* client, counselor, and other person, respectively. The summary and subscale scores of the Glasgow *Hikikomori* Scale (GHS) are indicated in the items of GHS: Daily Life and Self Care (DS), Occupational Role (OR), and Social Interaction (SI).

**Figure 2:**
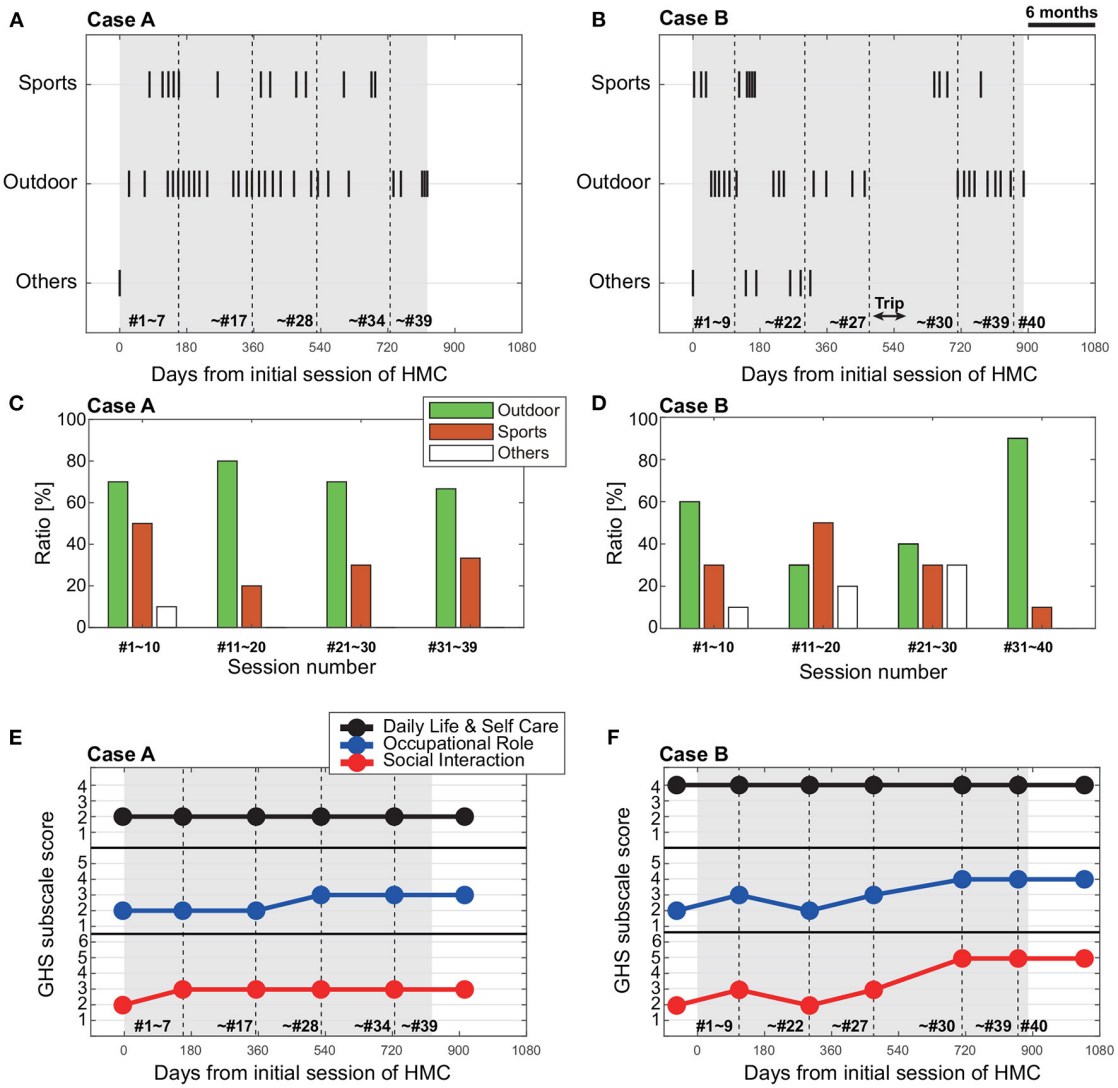
**(A, B)** Temporal plot of Human Movement Consultation (HMC) activity type of each case [**(A)** Case A, **(B)** Case B]. Sports and outdoor indicates the interpersonal sports and outdoor workouts, respectively. Others indicates the interview, volunteer, and tent building. The horizontal axis indicates the days from initial session of HMC. The black shaded area shows the period of HMC. The vertical dotted lines indicate the assessment dates of GHS. The numbers within the graphs indicate the HMC session number. **(C, D)** Ratio of activity type chosen during consecutive 9–10 sessions for each case [**(C)** Case A, **(D)** Case B]. **(E, F)** Time change of Glasgow *Hikikomori* Scale (GHS) subscale scores (Daily Life and Self Care, Occupational Role, and Social Interaction) for each case [**(E)** Case A, **(F)** Case B].

Case A participated in the following activities over the course of the HMC: outdoor workout (walking, running, and cycling: 25 sessions), interpersonal sports (table tennis, badminton, and playing catch-a-ball: 10 sessions), both outdoor workout and interpersonal sports (cycling and playing catch-a-ball or badminton: 3 sessions), and others (interview: 1 session). The distribution of these activities undertaken in the HMC sessions over the course of the HMC intervention are shown in Figure 2A. The ratio of activity types selected by the client for each quarter over the course of the intervention, is visualized in Figure 2C (the number of the final quarter of the sessions is 9, as the total sessions were 39). The most frequently chosen type of activity was the outdoor exercise. As for the interpersonal sports sessions (labeled “Sports” in Figure 2C), these were taken up initially, but less so in the second quarter, followed by an increase in the uptake toward the end of the programme. The pattern suggests that this client tried the interpersonal sports in the initial period, but they preferred the outdoor activities in the subsequent period, with the ratio becoming more even toward the end of treatment.

#### 3.1.2. GHS (social withdrawal) scores over time

The GHS summary and subscale scores, with dates of evaluation are shown in Table 2. Figure 2E shows the pattern of GHS scores over the course of the HMC intervention.

Before starting the HMC, the client described as Case A had received a score of 2 for each of the GHS subscales, Daily Life and Self Care, Occupational Role, and Social Interaction. The corresponding descriptions of the score for each subscale are as follows: Daily Life and Self Care was at the level of “restricted and diminished social life and daily living skills despite prompting and encouragement from others (e.g., parent/carer)”; Occupational Role was at the level of “only engages in age appropriate roles that are well below their ability and expected attainment with prompting and support of others (e.g., parent/carer )”; and Social Interaction was at the level of “has no social interaction at all outside of home and no interaction with family members (social interaction only online/via social media)”. The descriptions of scores for each subcategory is summarized in Table 1. During the HMC intervention, GHS scores never decreased, but increased at two time points. The first increase was after the 7th session, where the GHS subscale scores for Social Interaction had increased by one point (Figure 2E and Table 2), and was at the level of “engages in infrequent social interactions (less than one contact per week) by any means (including with family members and/or online/social media).” This increase suggests that the initiation of the HMC may have contributed to an improvement in the social interaction skills for this person. The second increase was after the 28th session, where the score for Occupational Role had increased one point (Figure 2E and Table 2), and was at the level of “engages in age appropriate roles that are well below their ability and expected attainment.” It was also around this session that an additional member had started joining the HMS sessions.

#### 3.1.3. Qualitative observations

During the initial interview (HMC session 1), Case A spoke to the HMC counselor regarding his condition and his low motivation. For example, he reported that “My body is too heavy” and “I'm always tired.” Further, he remarked that his sleep-wake cycle was reversed. During the interview, the counselor and the client reaffirmed that the primary aim of the HMC sessions was to recover his body awareness through physical activities tailored for his fitness level.

In the early sessions, decisions about what activities to do were led by the counselor and revolved around walking (session 2, 3), sometimes playing catch-a-ball (session 5–7), badminton (session 8), or table tennis (session 4). During these sessions, Case A would routinely remark, “Anything is okay. I cannot decide (on the type of activity).” During a table tennis session (session 4), he said, “It has been a long time since I moved my whole body like this.” In this session, counselor advised Case A to aim at keeping a steady and gentle rally to match his fitness level. During one of the cycling sessions (session 6), he shared with the counselor, “I was scared because I had the sensation that at any moment, I could bump into another person, or even a car.”

On the 8th session, he stated that “My days and nights are now closer to the regular cycle.” In the HMC sessions in which he walked with the counselor through the forest (session 9–13), he talked about his relationship with his parents. On the 13th session, he reported having positive feelings regarding his body recovery. He said, “I want to recover my physical strength.” Following this positive change, the counselor made adjustments for activity intensity and pace. In another table tennis session (session 14), he said, “I feel refreshed to perspire, as I had not experienced this for a very long time.” In the same session, he talked positively of his future, and remarked, “I want to return to university [in the following season].” He returned to university approximately 11 months after starting the HMC, after the 17th consultation session. The client expressed his wish to continue with the HMC, with the aim to recover his physical fitness. The counselor too had observed that his fitness had improved considerably since starting the HMC and they were able to engage in more intensive physical activities and cover greater distances by this point.

During the 23rd consultation session, Case A spoke about his struggles with making friends, which he had also mentioned to his psychiatrist. The psychiatrist suggested that he asked the HMC counselor for an additional member in the HMC activities. The client accepted the advice and accordingly, the HMC counselor introduced a female nurse to the activities in two sessions involving table tennis (session 27), and walk and run (session 28). For the subsequent three sessions, the client and counselor were joined by a male graduate student who study about *Hikikomori*, wherein they walked and ran together (session 29, 30, and 32). During these triadic sessions, it was observed that Case A began engaging in communication with the volunteer while avoiding physical proximity considered typical of social interaction. For example, he tended to walk behind the volunteer, presumably to avoid walking side-by-side with them but would initiate spontaneous conversations after an intense exertion (session 32). Approximately 21 months after the start of the HMCs (session 33), the client remarked on an improvement in his confidence in academic ability, “Now, I am able to achieve high grades in all disciplines, and I have gained confidence in my studies.” Prior to the onset of *Hikikomori*, Case A was a conscientious and high-performing student. By this point, he was able to achieve similar grades to those in the pre-onset period.

#### 3.1.4. Summary of the recovery process

In summary, the record across HMC sessions suggests that Case A's recovery process went through the following sequence of phases: (1) Initial indecision and lack of confidence in choosing activities, (2) Expressions of fear and lack of confidence (e.g., not feeling his body could handle the exercise, worried that he might hit a car); (3) Building trust in the counselor and talking about more personal issues (e.g., relationship with parents); (4) Growing physical confidence and enjoyment from challenging himself physically; (5) Increased confidence and other developments demonstrating agency (e.g., decision to return to university); (6) Development of motivation for other social capacity (e.g., willingness to try sessions with additional members in HMC activities); (7) Consolidation period with a full return to university role.

### 3.2. Case B

#### 3.2.1. The HMC sessions

Case B underwent 40 consultations (including one assessment interview), which amounted to approximately 73 h of consultation (Mean ± SD = 109.5 ± 88.6 min/session; Min = 30 min/session; Max = 360 min/session; note: measurement resolution is 30 min). The period between the initial and final sessions comprised 888 days (2 years and 158 days), and the mean interval between consecutive sessions was 22.7 days (SD = 29.4 days; Min = 3 days; Max = 187 days). The distribution of session dates and intervals were thus more varied for this client, who went through a pause during a relapse phase of extreme withdrawal (after session 17), and a long bike trip (after session 28). The overall breakdown of session distribution and details of activities during the HMC sessions are summarized in Table 3.

**Table 3:** HMC details of Case B (40 sessions).

**Number**	**Date**	**GHS score**	**Interval**	**Duration**	**Activity type**	**Location**	**Member**
	**(month-year)**	**Sum (DS, OR, SI)**	**(days)**	**(min)**			
	Oct-X	8 (4, 2, 2)					
1	Dec-X		–	30	Interview	Univ.	H, C
2	Dec-X		3	60	Tennis	Athletic ground in Univ.	H, C
3	Jan-X+1		19	60	Playing catch-a-ball	Athletic ground in Univ.	H, C
4	Jan-X+1		13	60	Table tennis	Gym in Univ.	H, C
5	Feb-X+1		14	150	Walking	Temple	H, C
6	Feb-X+1		10	60	Walking	Temple	H, C
7	Feb-X+1		11	60	Walking	Forest Park	H, C
8	Mar-X+1		14	60	Running	Forest Park	H, C
9	Mar-X+1		14	60	Running	Forest Park	H, C
	Apr-X+1	10 (4, 3, 3)					
10	Apr-X+1		19	60	Running	Forest Park	H, C
11	Apr-X+1		7	60	Soccer	Athletic ground in Univ.	H, C, Os
12	May-X+1		18	300	Volunteer	Lake Park	H, C, Os
13	May-X+1		3	60	Soccer	Athletic ground in Univ.	H, C, Os
14	May-X+1		7	60	Soccer	Athletic ground in Univ.	H, C, Os
15	May-X+1		7	60	Soccer	Athletic ground in Univ.	H, C, Os
16	May-X+1		7	60	Soccer	Athletic ground in Univ.	H, C, Os
17	Jun-X+1		4	300	Volunteer	Forest Park	H, C, Os
18	Jul-X+1		46	90	Running	Forest Park	H, C
19	Aug-X+1		15	90	Cycling	River Cycling Course	H, C
20	Aug-X+1		13	120	Cycling	Forest Cycling Course	H, C
21	Sep-X+1		17	180	Volunteer	Forest Park	H, C, Os
22	Oct-X+1		28	300	Volunteer	Forest Park	H, C, Os
	Oct-X+1	8 (4, 2, 2)					
23	Oct-X+1		26	90	Making a tent	Athletic ground in Univ.	H, C
24	Nov-X+1		9	300	Cycling, Making a tent	Forest and River Cycling Course	H, C
25	Dec-X+1		34	300	Cycling	River Cycling Course, Castle Park	H, C
26	Feb-X+2		70	360	Cycling	River Cycling Course	H, C
27	Mar-X+2		33	60	Running	Forest Park	H, C, O
	Apr-X+2	10 (4, 3, 3)					
28	Sep-X+2		187	60	Table tennis	Gym in Univ.	H, C
29	Oct-X+2		14	60	Hockey	Athletic ground in Univ.	H, C
30	Oct-X+2		21	60	Hockey	City Park	H, C
	Nov-X+2	13 (4, 4, 5)					
31	Nov-X+2		28	90	Cycling, Walking	Forest Park	H, C
32	Dec-X+2		17	90	Cycling, Walking	Forest Park	H, C
33	Dec-X+2		14	60	Cycling, Walking	Temple	H, C
34	Jan-X+3		14	60	Cycling, Walking	Temple	H, C
35	Jan-X+3		17	60	Badminton	Athletic ground in Univ.	H, C
36	Feb-X+3		18	60	Walking, Running	Forest Park	H, C
37	Mar-X+3		21	90	Walking, Running	Forest Park	H, C
38	Mar-X+3		14	90	Walking, Running	Forest Park	H, C
39	Apr-X+3		27	60	Running	City Park	H, C
	Apr-X+3	13 (4, 4, 5)					
40	May-X+3		35	90	Cycling, Walking	Forest Park	H, C
	Oct-X+3	13 (4, 4, 5)					
Mean ± SD			22.7 ± 29.4	109.5 ± 88.6			
Min			3	30			
Max			187	360			
Sum			888	4380 (73 h)			

The items of Interval and Duration indicates the days between two consecutive sessions and duration of HMC, respectively. H, C, and O indicate *Hikikomori* client, counselor, and other person, respectively. The summary and subscale scores of the Glasgow *Hikikomori* Scale (GHS) are indicated in the items of GHS: Daily Life and Self Care (DS), Occupational Role (OR), and Social Interaction (SI).

Case B performed the following activities in the course of HMC: outdoor workout (walking, running, and cycling: 23 sessions), interpersonal sports (tennis, table tennis, soccer, playing catch-a-ball: 12 sessions), and others (interview, volunteering, making a tent: 7 sessions). As with Case A, the chosen activity type in each session over the course of the HMC intervention are shown in Figure 2B, with the ratio of activity selections per 10 sessions shown in Figure 2D. From these figures, the outdoor workout was the preferred activity over the interpersonal sports, with the exception of the second block of the programme. The major factor for the higher ratio of participation in the interpersonal sports in the second phase was the social soccer sessions (Table 3; session 11, and 13–16). However, these participations were not his preferred choice over other activities in the following phase, instead, the other activities such as volunteering for tree cutting and practice building a tent for preparation of the planned trip, were increased. In the final phase after the long trip, the outdoor workout was strongly preferred. It is possible that the choice for these final sessions reflected the client's desire to talk about his thoughts around making a renewed start in his life.

#### 3.2.2. GHS (social withdrawal) scores over time

The GHS summary scores through the programme are shown in Table 3, and the subscale scores over the course of the intervention, are visualized in Figure 2F. Before the start of the HMC, the GHS subscale scores for Daily Life and Self Care was 4, and Occupational Role was 2, and Social Interaction was 2 (see Table 1 for scale description). During the HMC intervention, GHS scores fluctuated. The first point of increased score was recorded after the ninth session, where the GHS subscale scores for Occupational Role and Social Interaction was increased by one point (Figure 2F and Table 3). However, both the subscale scores went down after the 22nd session, then increased again after the 27th session. It is possible that the scores were improving as the intervention progressed, but became unstable after the relapse around the 17th session; with the HMC resumed, the scores gradually improved toward the end of the programme. The pattern may be contrasted with a seemingly more immediate improvement in scores after the long trip, where the subscale scores for Occupational Role and Social Interaction both increased by two points (Figure 2F and Table 3).

#### 3.2.3. Qualitative observations

In the assessment interview (session 1), Case B spoke about his sports experiences and that he was previously a triathlete. The counselor observed his advanced sporting skills and his high level of interests in sports from the start, when he played tennis (session 2), catch-a-ball (session 3), and table tennis (session 4). The client subsequently took initiative in requesting walking (sessions 5–7) and running (sessions 8–10), suggesting enthusiasm for the intervention. For example, when running, the client could lead the navigation of running routes (session 7) or identify new running routes using the GPS function on his smartphone (session 8–10).

Following these early positive outcomes, both the client's psychiatrist and the counselor suggested broadening of social dynamics in future sessions. The client agreed, and accordingly, the subsequent sessions included soccer (sessions 11, 13, 14, 15, and 16), and volunteering in forest maintenance (sessions 12 and 17). However, at approximately 6 months into the HMC (after session 17), the counselor could not get in touch with the client because the client reverted to a complete withdrawal state in his house. Approximately a month after the relapse, the counselor received an email requesting resumption of the HMC sessions. Following the client's return to the HMC, he shared his ambition for going on a long bike trip abroad (session 18). The content of the HMC around this point was then tailored to Case B's goal, which included various preparatory activities for the trip, such as long-distance cycling and practice putting up a tent (sessions 23 and 24). Around this time, he secured a part-time job to raise funds for his planned trip. Although Case B did not talk about the details of his reasons or a trigger of his *Hikikomori* period, estimating from the fact starting to execute his ambition after that period, it is considered that he would need time to think about himself in his home.

Approximately 16 months into receiving the HMC, Case B left for a cycling trip for three months. The HMC sessions were resumed upon his return, and he reported wanting to play sports such as table tennis (session 28) and field hockey (sessions 29 and 30). Around that time, the client told the counselor, “I decided to leave the university and start a new life.” During subsequent sessions involving walks through a forest (sessions 31, 32, and 36–38), he shared his thoughts and updates regarding his search for a new job. The HMC was terminated when he secured a new job position and confirmed his decision to leave the university.

A difference observed between Case A and B is that while Case A shared his emotions about his withdrawn state during the HMC, this was not the case for Case B. Case B rarely discussed his inner states with the counselor, including his withdrawal during the participation.

#### 3.2.4. Summary of the recovery process

Analysis of the phases in Case B's recovery process suggests that he went through the following sequence: (1) Trying outdoor workout and interpersonal sports activities with counselor (dyad interaction), (2) Challenging himself by allowing more complex social elements to enter the sessions (triad or larger group interaction; e.g., soccer and volunteering), (3) Social withdrawal period (with possible rumination and in depth analysis of life choices), (4) Re-connecting with the counselor to request the HMC to resume, (5) Sharing his ambition (bike long trip) with counselor and making preparations to execute it during the HMC (e.g., long-distance cycling and practice building a tent) as well as outside the intervention (securing a part-time job to raise fund), (6) Achievement of the bike trip (experience of outdoor workout alone and interacting with others abroad), (7) Making a decision to leave university and discussing his thoughts about starting new life.

## 4. Discussion

*Hikikomori* is an important problem but one for which we have very limited treatment and support options. By definition, severe *Hikikomori* sufferers do not seek help or engage with offers of support from the outside world. To make a meaningful difference to the lives of *Hikikomori* and their families there is a need to develop and test novel approaches that are both acceptable and show promise as effective treatments. This paper describes two cases where the HMC was successfully used with university students with moderate levels of *Hikikomori* behaviors. Applying this approach with less severely withdrawn patients provided a good “proof of concept” test of the HMC approach. Both the quantitative and qualitative evidence collected during this study suggest that the HMC procedures may be worth investigating in future studies.

The two cases receiving the HMC in this study were unique in their fitness level profiles as well as overall clinical presentation. Case A was a younger student whose athletic skills were under-developed and who was diagnosed with communication difficulties. In contrast, Case B was older, and had better sporting skills and fitness levels, and was without communication issues. In both cases, the social withdrawal levels assessed using behavioral indicators and the GHS showed improvements following receiving the HMC. The main improvements were seen in the domains of Occupational Role and Social Interaction and both people recovered sufficiently to re-engage with age-appropriate social roles and activities. However, the GHS domain of Daily Life and Self Care remained unchanged from start to end of HMC. One striking qualitative observation was that both people began to take ownership of their lives and made important decisions for their future (e.g., Case A decided to return to university and Cases B decided to pursue a different career path). Case B further demonstrated an improvement in his autonomy and capacity to identify personal goals and action plans when he embarked on his long-distance cycling trip.

Our results also provide interesting findings about the timeline of recovery during treatment with HMC. These observations raise issues worthy of investigation in future research. For instance, the Occupational Role and Social Interaction scores on the GHS showed improvement relatively early in the treatment sequence (between 7 and 10 h of contact) but these gains did not necessarily persist. In fact, both participants had episodes during the study period where they returned to extreme withdrawal, suggesting that the recovery process is non-linear and may involve periods of relapse until recovery is achieved. The challenge of reaching sustained improvement is also reflected in the long period of treatment contact of almost 3 years. This equated to 42 and 73 h of total contact for both participants and so it will be important for future refinements of the HMC approach to clarify parameters such as the optimal frequency and duration of contact. Also, because the GHS domain of Daily Life and Self Care remained unchanged from start to end of HMC it will be necessary to determine whether this aspect of the *Hikikomori* phenotype requires a different treatment approach or alternative outcome measurement strategy.

Although this is an uncontrolled observational study, the outcome of the HMC programmes for both people suggests that the intervention may have promoted important improvements in their symptoms of social withdrawal. In the following section, we consider the potential benefits of the HMC programme by activity types, and discuss potential mechanisms of action for the HMC on *Hikikomori* symptoms.

A decline in physical fitness levels can be one of the direct consequences of living in a state of *Hikikomori*. This was particularly highlighted in Case A, who demonstrated a diminished sense of physicality and overall low activity level prior to starting the HMC. For Case B, the HMC provided an opportunity to re-engage with activities he previously enjoyed, which appeared to prompt him to develop his own goal and achieve it through the cycling trip. One interpretation of these outcomes is that the HMC re-instilled a sense of agency in both recipients and helped them to switch from a passive mode to a more active and engaged life.

The results also show some possible interaction patterns worthy of investigation in future work. For instance, it seems that Case A spoke to the counselor unprompted more in the outdoor settings than in the social game activities. Likewise, it was also during an outdoor session that Case B shared his long-term plans for his life. One possibility is that greater interpersonal engagement during outdoor activities was facilitated by the spatial formation for joint attention, avoiding direct eye gaze (Figure 1A). Some mental health benefits have been reported for outdoor workout, known as “green exercise” (35), suggesting that there may be additional mental health gains for working out in nature (36). While it is not clear whether social aspects in the exercise is additionally beneficial (37), our outdoor sessions appeared to create important therapeutic opportunities for both people described in this study.

The outdoor activities and interpersonal sports, the two types of activities in the HMC, were not built in a hierarchical construct in the HMC. When comparing the chosen activity over time, the outdoor workout tended to be preferred over the interpersonal sports. Especially, Case A preferred the outdoor workout. There are several possible reasons for this, including that outdoor workouts are less competitive and have a lower emphasis on winning or losing. The outdoor activities may also present fewer opportunities for meeting other persons, and it may have been easier to talk to the counselor because of the reduced face-to-face contact. Some of the outdoor tasks may have helped recover body awareness and improve physical strength through aerobic movement. Although we did not record physiological data, Case A recovered his physical awareness and improved physical strength gradually such that he became fit enough to run from the 12th session. In contrast, the main reason Case B preferred the outdoor workout might simply be that he liked to run and bike (because he was a triathlete) and this was expressed in his ambition to take a long bike trip. However, after a trip, he seemed to prefer the outdoor workout, for the reason of making opportunities to talk about his future with others the counselor. Although the two cases were different types of *Hikikomori*, the outdoor workouts play multiple effectiveness according to the *Hikikomori* state and individual particular. This is considered the reason that outdoor workout trend to choose than interpersonal sports.

The HMC programme reported in this study emphasizes empowering clients and giving them choices. While clients such as Case A may not have been able to make decisions about activities initially, it was observed that client-led sessions provided them with a basis for exploring their sense of self, nurturing autonomy, and providing motivation in their participation in the programme. Further, and more importantly, the positive effects observed during the HMC sessions appeared to generalize, to relate to important personal development for both people. During the course of the HMC sessions, both cases showed a therapeutically meaningful role reversal such that the client took initiative in the direction and content of the sessions. We suggest that such an outcome was in part due to encouraging the clients to develop a sense of agency in a safe and non-pressured context. Likewise, the sessions permitted exploration of their personal goals and aspirations. As the sessions progressed, they contributed toward the clients' pursuit of their long-term goals and desires. Although it is unlikely that the HMC programme alone was responsible for these positive changes and the data from the study cannot address the causality of this possibility, providing graded non-threatening opportunities for interpersonal contact does present a plausible therapeutic ingredient. The sessions may have facilitated a meaningful and safe context in which the clients were able to explore possibilities and emerging aspirations with the help of the counselor. This was made possible by the principle of the HMC in allowing flexibility for both clients and the session provider, so that appropriate levels of challenges may be introduced, without placing any pressure on the clients to accept them.

Our experience with these two cases also generated new ideas about how to plan the intensity and frequency of the physical activity in HMC sessions. When starting HMC or after a period of lost contact due to *Hikikomori* withdrawal, the intensity should be titrated to avoid participants being overwhelmed with physical load and/or mental stress. Planning for this could include collaboration with the wider care team such as timing sessions to be interspersed with consultation with the psychiatrist. Agreeing to spread initial contacts on a less intensive schedule (e.g., every 2 weeks) may also help recipients become familiar with the HMC process at a manageable pace.

The two cases reported in this study support the view that a specific difficulty of this group may relate more to interpersonal relationships (38) rather than broader social or communication difficulties. This position proposes that *Hikikomori* ought to be distinguished from other disorders such as autism or social anxiety, amongst the ongoing debate around co-morbidity of *Hikikomori* (1). While the current study is not suitable for addressing the query, future research should assess more diverse client profiles for the HMC program.

We have presented the first early phase investigation of how to treat *Hikikomori* using long-term exercise therapy that also promotes autonomy and empowerment. While the outcomes of the two clients appear positive, there is more to be done in examining the effective aspects of the HMC intervention. A number of questions about the key therapeutic ingredients are worthy of investigation in next phase studies. For instance, measuring physical parameters such as blood pressure, heart rate, and number of daily steps taken would improve the value of future studies by clarifying how much the HMC approach affects physical health. Moreover, it may have been helpful in that someone outside of the standard psychiatric therapy relationship provided a listening ear and an attuned mind to the client. It is also possible that the physical fitness gains and improved mastery that came with the physical activities themselves contributed to the outcomes. The range of new *Hikikomori* assessment scales and observational measures released since we commenced the current study opens up a number of other measurement options that should be considered in next phase studies designed to support recovery from extreme social withdrawal. Future studies should also be designed to further analyze and test the active ingredients of the HMC programme. It is equally important to explore more *Hikikomori* client profiles and to further build the evidence base for studying the efficacy of the HMC intervention. Further data will also make important contribution toward studying the feasibility and acceptability of the HMC programme by *Hikikomori* as a population group. One of the critical steps here would concern studying suitable clinical profiles for offering the HMC, as well as identifying the optimal timing for starting and terminating the HMC. Ultimately, controlled clinical trials with sufficiently powered samples would be able to address these outstanding questions. They would also help uncover an ideal role for the HMC as part of providing support for this group, estimated to be substantial in numbers across the globe, and yet suffering unseen by society, while causing much distress to themselves and their loved ones.

## Data availability statement

The original contributions presented in the study are included in the article/supplementary material, further inquiries can be directed to the corresponding author.

## Ethics statement

The studies involving human participants were reviewed and approved by Research Center of Health, Physical Fitness and Sports, Nagoya University, Japan. The patients/participants provided their written informed consent to participate in this study. Written informed consent was obtained from the individual(s) for the publication of any potentially identifiable images or data included in this article.

## Author contributions

KY, TF, YY, MR, and HM conceived and designed research and wrote the paper. KY and TF collected and analyzed data. All authors contributed to the article and approved the submitted version.

## Footnotes

^1^There has been increase in the number of scales published to assess *Hikikomori* in the past few years (33, 34) but these were not available when we designed and commenced the current study.
